# Galectin-3 Beyond Conventional Cardiac Biomarkers and Its Incremental Prognostic Value in Heart Failure: A Systematic Review

**DOI:** 10.7759/cureus.109592

**Published:** 2026-05-25

**Authors:** Vaishnavi Hemdev, Anushka Rani, Ahsan Qadeer, Parvati Sunjay Kumar, Fnu Nancy, Shabir Zarif

**Affiliations:** 1 Internal Medicine, University of York, York, GBR; 2 Internal Medicine, Peoples University of Medical and Health Sciences for Women, Nawabshah, PAK; 3 Internal Medicine, King Edward Medical University, Lahore, PAK; 4 Internal Medicine, Nishtar Medical University, Multan, PAK

**Keywords:** acute heart failure, biomarkers, galectin-3, heart failure, hfpef, myocardial fibrosis, natriuretic peptides, prognosis, risk stratification

## Abstract

Heart failure is a heterogeneous clinical syndrome in which accurate risk stratification remains challenging despite the widespread use of natriuretic peptides and cardiac troponins. Galectin-3, a biomarker of inflammation and myocardial fibrosis, has emerged as a potential complementary prognostic marker; however, its incremental value beyond established biomarkers remains incompletely defined. This systematic review synthesizes primary clinical evidence evaluating the prognostic role of galectin-3 in adult heart failure populations, with a specific focus on its additive value beyond conventional cardiac biomarkers. A comprehensive literature search identified seven high-quality primary studies encompassing community-based cohorts, ambulatory chronic heart failure, heart failure with preserved ejection fraction, acute decompensated heart failure, and post-discharge populations. Across these studies, galectin-3 demonstrated incremental prognostic value for adverse outcomes, including mortality and heart failure-related hospitalization, particularly in fibrosis-dominant phenotypes such as heart failure with preserved ejection fraction and acute or recently decompensated heart failure. In contrast, its prognostic utility was attenuated in well-treated ambulatory patients with chronic heart failure with reduced ejection fraction after adjustment for natriuretic peptides. Importantly, studies incorporating serial galectin-3 measurements consistently demonstrated stronger associations with adverse outcomes than single baseline assessments, highlighting the value of longitudinal biomarker evaluation. Overall, the available evidence supports a phenotype-specific and dynamic role for galectin-3 as a complementary prognostic biomarker that reflects myocardial remodeling rather than hemodynamic stress alone. These findings refine the clinical positioning of galectin-3 and underscore its potential utility in risk stratification and disease monitoring, while emphasizing the need for prospective studies to evaluate galectin-3-guided management strategies.

## Introduction and background

Heart failure (HF) is a leading cause of morbidity, mortality, and healthcare utilization worldwide. Despite advances in pharmacological therapy and device-based interventions, clinical outcomes remain poor, largely because of the heterogeneity of underlying disease mechanisms and persistent limitations in risk stratification [1]. Accurate prognostic assessment is essential for guiding treatment decisions, identifying high-risk patients, and optimizing follow-up strategies across the spectrum of HF [2].

Cardiac biomarkers play a central role in the management of HF. Natriuretic peptides, including B-type natriuretic peptide (BNP) and N-terminal pro-B-type natriuretic peptide (NT-proBNP), reflect myocardial wall stress and are widely used for diagnosis and prognostication, whereas cardiac troponins indicate ongoing myocardial injury [3,4]. However, these biomarkers primarily capture hemodynamic stress and myocyte necrosis, providing limited insight into other key pathological processes, such as inflammation, fibrosis, and structural remodeling, which are major contributors to HF progression [5].

Galectin-3, a β-galactoside-binding lectin secreted predominantly by activated macrophages and upregulated within fibrotic and remodeling regions of the myocardium, particularly the left ventricle in pressure- and volume-overload states, has emerged as a biomarker of inflammatory and fibrotic activity in HF [6]. It promotes fibroblast activation, collagen deposition, and adverse ventricular remodeling, thereby linking immune activation to myocardial fibrosis [7]. Elevated galectin-3 levels have been associated with worse outcomes in both acute and chronic HF, including increased mortality and rehospitalization. Importantly, these associations have frequently remained significant after adjustment for established biomarkers such as BNP, NT-proBNP, and cardiac troponin, suggesting that galectin-3 may provide complementary pathophysiological information rather than merely duplicating existing risk markers [8].

The objective of this systematic review is to evaluate whether galectin-3 provides incremental prognostic value beyond conventional cardiac biomarkers, including cardiac troponins and natriuretic peptides, for predicting HF progression and adverse clinical outcomes such as all-cause mortality and HF-related hospitalization in adult patients with HF.

## Review

Materials and methods

Study Design and Reporting Framework

This systematic review was conducted in accordance with the Preferred Reporting Items for Systematic Reviews and Meta-Analyses (PRISMA) guidelines [9]. The aim of the review was to synthesize primary clinical evidence evaluating the incremental prognostic value of galectin-3 beyond established cardiac biomarkers, particularly cardiac troponins and natriuretic peptides, in patients with HF. A predefined protocol guided study selection, data extraction, and risk of bias assessment to ensure methodological rigor and transparency.

Research Question and Population, Intervention/Exposure, Comparator, and Outcome (PICO) Framework

The research question was formulated using the PICO framework [10]. The population comprised adult patients with HF across the disease spectrum, including community-based individuals at risk of incident HF, ambulatory patients with chronic HF, patients with HF with preserved ejection fraction (HFpEF), recently hospitalized patients with HF, and patients with acute decompensated HF.

The exposure of interest was plasma galectin-3 measured at baseline and, when available, through serial assessments. Comparator biomarkers included established cardiac biomarkers, specifically BNP, NT-proBNP, and cardiac troponins. The primary outcomes of interest were all-cause mortality and HF-related hospitalization. Secondary outcomes included incident HF, composite clinical endpoints, cardiac transplantation, ventricular assist device implantation, and near-term rehospitalization.

Literature Search Strategy

A comprehensive literature search was conducted using PubMed/MEDLINE, Embase, and the Cochrane Central Register of Controlled Trials (CENTRAL) to identify relevant studies from database inception to the most recent available publications. Search terms included combinations of keywords and Medical Subject Headings (MeSH) related to “galectin-3,” “heart failure,” “prognosis,” “mortality,” “hospitalization,” “BNP,” “NT-proBNP,” and “troponin.”

The search strategy was designed to capture studies explicitly evaluating galectin-3 in prognostic models that included established cardiac biomarkers. Reference lists of eligible studies were also manually screened to identify additional relevant articles. Only studies published in the English language were considered.

Study Selection and Eligibility Criteria

Studies were eligible for inclusion if they were primary clinical studies, including prospective observational cohorts or randomized controlled trial biomarker substudies, that evaluated the prognostic association between galectin-3 and clinical outcomes in HF populations. Included studies were required to report multivariable analyses adjusted for at least one established cardiac biomarker, such as BNP, NT-proBNP, or cardiac troponin.

Studies focusing solely on diagnostic performance, cross-sectional associations without longitudinal outcomes, editorials, narrative reviews, and meta-analyses were excluded. Two reviewers independently screened titles and abstracts, followed by full-text assessment for eligibility. Discrepancies were resolved by consensus.

Data Extraction and Synthesis

Data were extracted using a standardized data extraction form developed a priori. Extracted variables included study characteristics, patient population, and HF phenotype, sample size, galectin-3 measurement strategy, comparator biomarkers, outcomes assessed, duration of follow-up, covariates included in multivariable models, and evidence of incremental prognostic value. Given the heterogeneity in study populations, outcome definitions, biomarker measurement methods, and statistical approaches, a qualitative narrative synthesis was performed rather than a quantitative meta-analysis.

Risk of Bias Assessment

Risk of bias was assessed at the study level using tools appropriate to the study design. Observational cohort studies were evaluated using the Newcastle-Ottawa Scale [11], which assesses selection, comparability, and outcome domains. Randomized controlled trial biomarker substudies were evaluated using the Cochrane Risk of Bias 2 (RoB 2) tool [12], applied conservatively to biomarker analyses. Each study was categorized as having low, moderate, or high risk of bias based on domain-level judgments.

Statistical Considerations

As this review synthesized previously published results, no new statistical analyses were performed. Effect estimates, including hazard ratios, odds ratios, confidence intervals, and discrimination metrics reported by individual studies, were interpreted descriptively. Particular emphasis was placed on whether galectin-3 retained prognostic significance after adjustment for established biomarkers and whether it improved risk discrimination or reclassification.

Results

Study Selection Process

**Figure 1:**
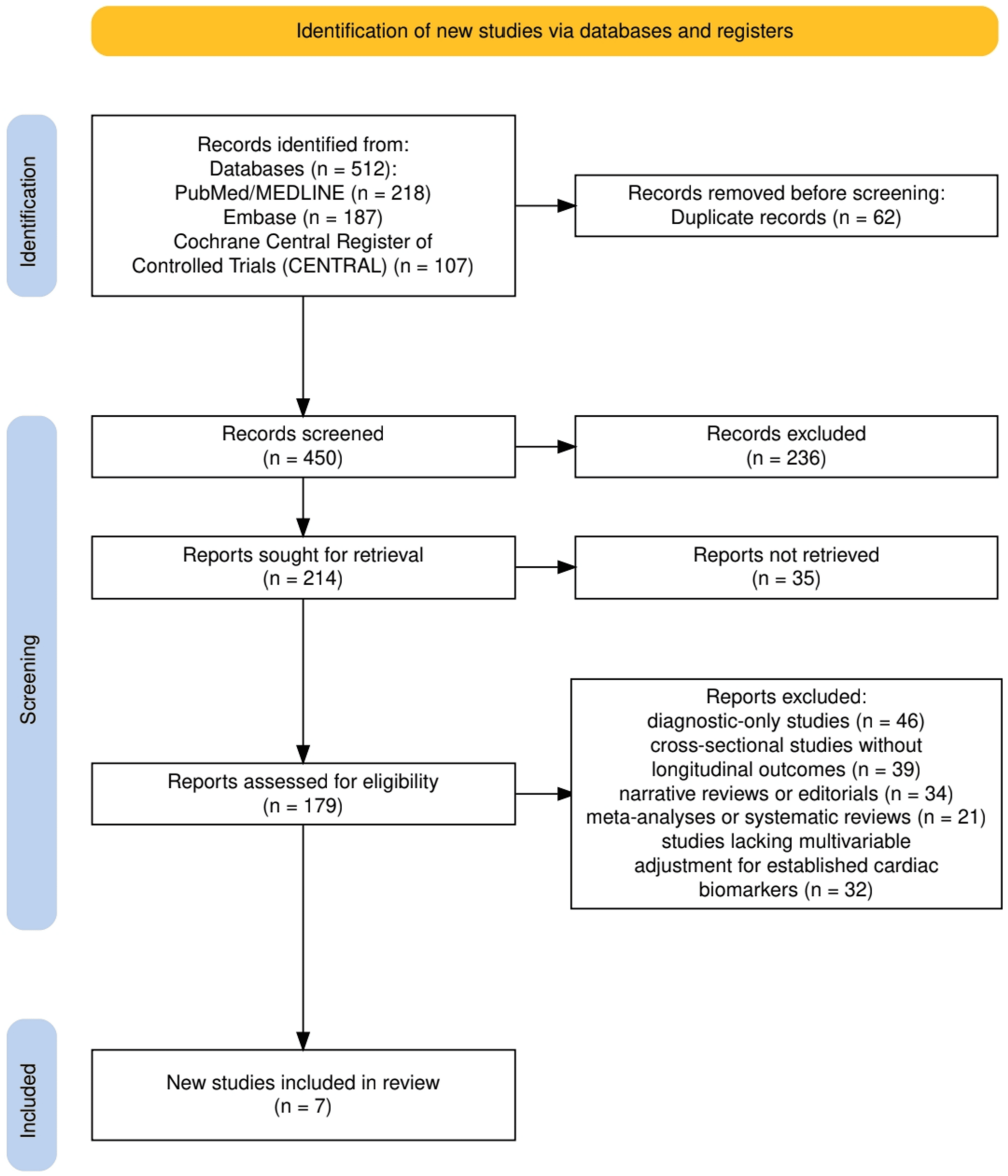
PRISMA flow diagram of study selection process for inclusion in the systematic review. PRISMA, Preferred Reporting Items for Systematic Reviews and Meta-Analyses

The study selection process is summarized in Figure 1. A total of 512 records were identified through database searching, including PubMed/MEDLINE (n = 218), Embase (n = 187), and the CENTRAL (n = 107). After removal of 62 duplicate records, 450 studies underwent title and abstract screening, of which 236 were excluded for not meeting the inclusion criteria. Full texts were sought for 214 reports, with 35 reports not retrieved, leaving 179 studies assessed for eligibility. Of these, 172 studies were excluded due to diagnostic-only design, lack of longitudinal outcomes, narrative or review format, absence of multivariable adjustment for established cardiac biomarkers, or duplication of evidence. Ultimately, seven studies met all eligibility criteria and were included in the qualitative synthesis. The stepwise screening and exclusion process ensured inclusion of high-quality primary evidence directly addressing the incremental prognostic value of galectin-3 beyond conventional cardiac biomarkers.

Characteristics of the Selected Studies

The key characteristics of the included studies are summarized in Table 1. The seven studies comprised a total of 7,568 participants and represented a broad spectrum of HF populations, including community-based individuals at risk for incident HF, ambulatory chronic HF cohorts, patients with HFpEF, recently hospitalized HF patients, and those presenting with acute or decompensated HF. Sample sizes ranged from 115 to 3,353 participants, with follow-up durations extending from 30 days to over 11 years. Galectin-3 was measured at baseline in all studies, while two studies additionally incorporated serial galectin-3 assessments, enabling evaluation of temporal changes in biomarker levels. All included studies evaluated galectin-3 in multivariable prognostic models adjusted for established cardiac biomarkers, most commonly BNP or NT-proBNP, and in some cases troponin. Clinical outcomes primarily included all-cause mortality and HF-related hospitalization, with several studies reporting composite endpoints or incident HF. Importantly, galectin-3 cut-off values were not standardized across studies, with investigators using different thresholds, categorical stratifications, or continuous risk models. Collectively, the included studies were heterogeneous in design and population but consistently addressed the prognostic role of galectin-3 beyond conventional biomarkers, providing a comprehensive evidence base for qualitative synthesis.

**Table 1:** Characteristics of included primary studies evaluating the incremental prognostic value of galectin-3 beyond conventional cardiac biomarkers in diverse heart failure populations. BNP, B-type natriuretic peptide; ED, emergency department; EF, ejection fraction; Gal-3, galectin-3; HF, heart failure; HFpEF, heart failure with preserved ejection fraction; HFrEF, heart failure with reduced ejection fraction; HR, hazard ratio; IQR, interquartile range; LVEF, left ventricular ejection fraction; NT-proBNP, N-terminal pro-B-type natriuretic peptide; NYHA, New York Heart Association; RCT, randomized controlled trial; SD, standard deviation; VAD, ventricular assist device

Study (Year)	Population and HF Type	Sample Size	Galectin-3 Measurement	Comparator Biomarkers	Outcomes Assessed	Follow-Up Duration	Key Adjustments (Multivariable Models)	Main Findings (Incremental Value)
Ho et al., 2012 [13]	Community-based adults (no HF at baseline); incident HF risk	3,353	Baseline plasma galectin-3 (log-transformed; per 1 SD increase)	BNP	Incident heart failure; all-cause mortality	Mean 11.2 years	Age, sex, clinical risk factors, BNP	Galectin-3 independently predicted incident HF (HR 1.23 per SD increase) and all-cause mortality after adjustment for BNP; addition of Gal-3 resulted in a minor improvement in risk reclassification
Edelmann et al., 2015 (Aldo-DHF) [14]	Stable HFpEF patients (NYHA II-III; LVEF ≥50%)	422	Baseline galectin-3 and serial measurements at 6 and 12 months	NT-proBNP	All-cause death or hospitalization	12 months	NT-proBNP, treatment arm (spironolactone vs placebo), clinical covariates	Rising galectin-3 levels over time were independently associated with increased risk of death or hospitalization, providing prognostic value beyond NT-proBNP; baseline galectin-3 correlated with worse functional status and quality of life
French et al., 2016 [15]	Ambulatory chronic HF (HFrEF, HFpEF, recovered EF)	1,385	Baseline plasma galectin-3 (per doubling of concentration)	BNP, troponin I, soluble ST2	All-cause mortality, cardiac transplantation, ventricular assist device implantation	Up to 5 years	Age, sex, clinical covariates, comparator biomarkers	Higher galectin-3 levels were independently associated with adverse outcomes; galectin-3 demonstrated superior long-term risk discrimination in HFpEF compared with BNP and troponin, supporting incremental prognostic value beyond established biomarkers
Felker et al., 2012 (HF-ACTION) [16]	Ambulatory chronic HFrEF patients (exercise trial cohort)	895	Baseline plasma galectin-3 (per 3-ng/mL increase)	NT-proBNP	Hospitalization-free survival	Median ~2.5 years	Age, sex, renal function, functional capacity, NT-proBNP, clinical covariates	Galectin-3 was associated with worse outcomes in unadjusted analyses; however, its prognostic significance was attenuated and no longer independent after adjustment for NT-proBNP, suggesting limited incremental value beyond natriuretic peptides in ambulatory HFrEF
Meijers et al., 2014 [17]	Recently hospitalized HF patients (mixed EF)	902	Baseline plasma galectin-3 measured during index admission (categorical cut-off ≥17.8 ng/mL)	BNP	HF rehospitalization; composite of all-cause mortality and rehospitalization	30, 60, 90, and 120 days post-discharge	Age, sex, NYHA class, renal function, LVEF, BNP	Elevated galectin-3 independently predicted near-term HF rehospitalization after adjustment for BNP; addition of galectin-3 significantly improved risk reclassification for rehospitalization and fatal events, demonstrating incremental prognostic value beyond natriuretic peptides
van Vark et al., 2017 (TRIUMPH) [18]	Hospitalized acute heart failure patients	496	Baseline and repeated galectin-3 measurements (median 4.3 per patient over 1 year)	NT-proBNP (baseline and serial)	Composite of all-cause mortality and HF rehospitalization	Median 325 days (IQR 85-401)	Clinical covariates, baseline and serial NT-proBNP	Serial galectin-3 measurements were strongly associated with adverse outcomes; repeated galectin-3 provided independent and incremental prognostic value beyond both baseline and serial NT-proBNP, whereas baseline galectin-3 alone showed only a weak association
Shah et al., 2010 [19]	Emergency department patients with acute dyspnea; subgroup with acute decompensated HF	115	Baseline plasma galectin-3 measured at admission (median-based stratification)	NT-proBNP; echocardiographic risk markers	All-cause mortality	Up to 4 years	Age, renal function, NT-proBNP, and detailed echocardiographic parameters	Elevated admission galectin-3 levels independently predicted long-term mortality in patients with acute decompensated HF, providing prognostic information beyond echocardiographic markers and NT-proBNP

Quality Assessment

Overall, the included studies demonstrated low to moderate risk of bias, reflecting generally robust methodological quality across the primary evidence base (Table 2). Large prospective cohort studies, including the community-based and ambulatory HF cohorts, were judged to be at low risk of bias, owing to well-defined populations, standardized galectin-3 measurements obtained prior to outcome occurrence, and comprehensive multivariable adjustment for key confounders such as age, renal function, and natriuretic peptides. Randomized controlled trial biomarker substudies similarly exhibited low risk of bias, supported by randomized trial frameworks, blinded outcome assessment, and standardized follow-up procedures. A small number of studies were rated as having moderate risk of bias, primarily due to potential selection bias associated with single-center designs, pooled cohort heterogeneity, or residual confounding inherent to observational analyses. Nevertheless, outcome assessment across studies was largely objective and consistently defined, and no evidence of selective reporting was identified. Collectively, the risk of bias profile supports the reliability of the synthesized findings while acknowledging unavoidable limitations related to study design and population heterogeneity.

**Table 2:** Risk of bias assessment of included studies evaluating the incremental prognostic value of galectin-3 beyond conventional cardiac biomarkers in heart failure. BNP, B-type natriuretic peptide; ED, emergency department; HF, heart failure; NT-proBNP, N-terminal pro-B-type natriuretic peptide; RCT, randomized controlled trial; RoB 2, Risk of Bias 2

Study (Year)	Study Type	Risk of Bias Tool Used	Selection Bias	Measurement Bias	Confounding Bias	Outcome Assessment Bias	Overall Risk of Bias	Justification
Ho et al., 2012 [13]	Prospective community cohort	Newcastle-Ottawa Scale	Low	Low	Low	Low	Low risk	Large, well-characterized cohort with long follow-up; galectin-3 measured prior to outcomes; robust multivariable adjustment including BNP
Edelmann et al., 2015 (Aldo-DHF) [14]	RCT biomarker analysis	Cochrane RoB 2	Low	Low	Low	Low	Low risk	Randomized trial framework; standardized biomarker measurements; outcomes assessed independently of galectin-3; appropriate adjustment for NT-proBNP
French et al., 2016 [15]	Prospective ambulatory HF cohort	Newcastle-Ottawa Scale	Low	Low	Moderate	Low	Low-moderate risk	Well-phenotyped cohort with long follow-up; potential residual confounding due to mixed HF phenotypes despite extensive biomarker adjustment
Felker et al., 2012 (HF-ACTION) [16]	RCT biomarker substudy	Cochrane RoB 2	Low	Low	Low	Low	Low risk	Trial-level adjudication and standardized outcome assessment; neutral findings reduce risk of selective reporting
Meijers et al., 2014 [17]	Pooled prospective cohorts	Newcastle-Ottawa Scale	Moderate	Low	Low	Low	Low-moderate risk	Pooled analysis across cohorts introduces heterogeneity; strong statistical adjustment and reclassification analyses mitigate bias
van Vark et al., 2017 (TRIUMPH) [18]	Prospective acute HF cohort	Newcastle-Ottawa Scale	Low	Low	Low	Low	Low risk	Repeated biomarker measurements analyzed using joint modeling; adjustment for serial NT-proBNP minimizes confounding
Shah et al., 2010 [19]	Prospective ED cohort	Newcastle-Ottawa Scale	Moderate	Low	Moderate	Low	Moderate risk	Small sample size and single-center design increase selection bias; however, objective outcomes and detailed adjustment reduce measurement bias

Discussion

Overview of Principal Prognostic Patterns

Across the included studies, a consistent pattern emerged in which galectin-3 provided incremental prognostic information beyond conventional cardiac biomarkers, although its clinical utility appeared highly context- and phenotype-dependent. In community-based and hospitalized HF populations, Ho et al. [13] and Meijers et al. [17] demonstrated that elevated galectin-3 levels remained independently associated with incident HF, mortality, and near-term rehospitalization after adjustment for BNP or NT-proBNP, indicating additive prognostic value beyond hemodynamic stress markers.

This incremental utility was most pronounced in fibrosis-dominant HF phenotypes, particularly HFpEF and acute or recently decompensated HF, as shown by Edelmann et al. [14], French et al. [15], and Shah et al. [19], in which galectin-3 consistently outperformed or complemented natriuretic peptides in predicting adverse outcomes. In contrast, among well-treated ambulatory patients with chronic HF with reduced ejection fraction (HFrEF), Felker et al. [16] reported attenuation of the prognostic significance of galectin-3 after adjustment for NT-proBNP, underscoring important phenotype-specific limitations.

Importantly, studies incorporating serial galectin-3 assessment, most notably that of van Vark et al. [18], demonstrated substantially stronger associations with mortality and HF rehospitalization than single baseline measurements. These findings support the concept that galectin-3 may function more effectively as a dynamic biomarker of progressive myocardial fibrosis rather than as a static risk marker.

Mechanistic Basis for the Incremental Prognostic Value of Galectin-3

The incremental prognostic value of galectin-3 may be explained by its reflection of pathobiological processes distinct from those captured by conventional cardiac biomarkers [20]. Galectin-3 is secreted predominantly by activated macrophages and functions as a key mediator of fibroblast activation, collagen deposition, and extracellular matrix remodeling, placing it at the center of myocardial fibrotic signaling [21].

In contrast, natriuretic peptides such as BNP and NT-proBNP primarily reflect myocardial wall stress and intracardiac filling pressures, whereas cardiac troponins indicate active myocyte injury or necrosis. Importantly, fibrotic remodeling is a slowly progressive and often clinically silent process that may continue despite apparent hemodynamic stability or symptomatic improvement, particularly in chronic or compensated HF states [22].

This mechanistic divergence helps explain why galectin-3 retained prognostic significance in several studies even when natriuretic peptide levels plateaued or lost discriminative capacity, as demonstrated by Ho et al. [13], Shah et al. [19], and Edelmann et al. [14]. By capturing the cumulative burden of inflammatory and fibrotic activity, galectin-3 may provide complementary insight into disease progression that is not adequately reflected by markers of wall stress or myocardial injury alone.

Phenotype-Specific Utility of Galectin-3 in Heart Failure

A critical insight from this review is that the prognostic utility of galectin-3 is not uniform across HF phenotypes, but instead aligns closely with the dominant underlying pathophysiology. In HFpEF, where myocardial fibrosis, inflammation, and microvascular dysfunction play central roles, galectin-3 demonstrated its strongest incremental value [23]. Studies by Edelmann et al. [14] and French et al. [15] showed that galectin-3 independently predicted adverse outcomes and, in some cases, outperformed BNP and troponin in long-term risk discrimination. Similarly, in acute and recently decompensated HF, Shah et al. [19], Meijers et al. [17], and van Vark et al. [18] reported robust associations between galectin-3 and long-term mortality or rehospitalization, reflecting the prognostic impact of active remodeling triggered by acute injury or volume overload. In contrast, among ambulatory patients with chronic HFrEF enrolled in the HF-ACTION trial, Felker et al. observed attenuation of galectin-3’s prognostic value after adjustment for NT-proBNP, suggesting that in advanced systolic dysfunction dominated by hemodynamic stress, natriuretic peptides may already capture most clinically relevant risk [16]. These findings shift the clinical question from whether galectin-3 is useful to where it is most informative.

Static Versus Dynamic Biomarker Assessment

One of the most novel contributions of this review is the reframing of galectin-3 as a dynamic biomarker of disease progression rather than a static risk indicator. While baseline galectin-3 levels were modest predictors of outcome in several cohorts, studies incorporating serial measurements demonstrated substantially stronger prognostic associations [24]. In the TRIUMPH cohort, van Vark et al. [18] showed that rising galectin-3 levels over time were independently associated with mortality and HF rehospitalization, even after adjustment for both baseline and serial NT-proBNP, whereas baseline galectin-3 alone showed weaker predictive value. Similar longitudinal patterns were observed in HFpEF populations studied by Edelmann et al. [14], reinforcing the concept that temporal changes in galectin-3 reflect ongoing fibrotic activity and structural remodeling. This contrasts sharply with natriuretic peptides, which fluctuate rapidly in response to changes in loading conditions and therapy. By capturing a slower, cumulative biological process, serial galectin-3 assessment may offer a unique window into progressive myocardial remodeling, representing a shift from episodic risk assessment toward continuous disease monitoring [25].

Clinical Implications and Translational Relevance

The findings of this review support a selective and complementary clinical role for galectin-3 in HF risk assessment rather than routine universal testing. Galectin-3 appears most useful for risk stratification in high-risk settings, particularly following hospitalization for acute or decompensated HF, where studies by Meijers et al. [17] and van Vark et al. [18] demonstrated improved prediction of rehospitalization and mortality beyond natriuretic peptides. In HFpEF, elevated or rising galectin-3 levels may help identify patients with active fibrotic remodeling, a subgroup often inadequately characterized by traditional biomarkers. Importantly, current evidence supports the use of galectin-3 primarily for monitoring disease progression and long-term risk, rather than for guiding therapeutic decisions, as no interventional strategies have yet been validated on the basis of galectin-3 levels alone [26]. However, future integration of galectin-3 into multi-biomarker and phenotype-guided strategies may eventually influence surveillance intensity, follow-up planning, and individualized therapeutic decision-making. This conservative positioning aligns galectin-3 with emerging paradigms of longitudinal risk assessment while avoiding premature clinical implementation.

Limitations of the Available Evidence

Several limitations should be considered when interpreting the findings of this review. Substantial heterogeneity exists across included studies with respect to patient populations, HF phenotypes, galectin-3 assay methods, comparator biomarkers, and outcome definitions, precluding quantitative synthesis and limiting direct comparability. Additionally, there is a lack of standardized galectin-3 cut-off values, with studies employing varying thresholds, transformations, and longitudinal change metrics. The predominance of observational study designs introduces the possibility of residual confounding despite comprehensive multivariable adjustment, and biomarker substudies of randomized trials were not specifically designed to evaluate galectin-3-guided strategies. Finally, the absence of therapeutic trials using galectin-3 to guide treatment decisions limits the ability to draw causal or actionable conclusions regarding clinical intervention.

Future Directions and Research Priorities

Future research should focus on moving galectin-3 from a prognostic marker toward a tool for structured disease monitoring. Prospective studies evaluating galectin-3-guided surveillance strategies, particularly in HFpEF and post-discharge acute HF populations, may clarify its role in identifying patients at risk for progressive remodeling. Integration of galectin-3 with imaging-based fibrosis assessments, such as cardiac magnetic resonance-derived extracellular volume or late gadolinium enhancement, could enhance phenotypic characterization and mechanistic understanding, as prior studies have demonstrated associations between galectin-3 and structural remodeling or myocardial aging [19,27]. Additionally, galectin-3 is likely to be most informative when incorporated into multi-biomarker panels capturing complementary pathways involving wall stress, myocardial injury, inflammation, and fibrosis [8,20,22]. Importantly, biomarker predominance may vary across different stages and phenotypes of HF, and elevated galectin-3 levels may also reflect extracardiac fibrotic or inflammatory processes, including chronic kidney disease, resistant hypertension, and systemic infiltrative disorders such as amyloidosis [20,23,24]. Future studies should therefore focus on improving phenotype-specific interpretation and distinguishing cardiac remodeling from systemic fibrosis or renal dysfunction. Finally, growing interest in anti-fibrotic therapies targeting galectin-3-mediated pathways, including experimental galectin-3 inhibitors and fibrosis-modulating strategies, highlights the potential for future biomarker-guided therapeutic approaches [6-8]. Ultimately, interventional trials targeting fibrotic pathways in patients stratified by elevated or rising galectin-3 levels will be necessary to determine whether this biomarker can transition from prognostic indicator to a clinically actionable tool.

## Conclusions

This systematic review demonstrates that galectin-3 provides context-dependent and biologically complementary prognostic information beyond established cardiac biomarkers in HF, with its greatest value observed in fibrotic-dominant phenotypes such as HFpEF and acute or recently decompensated HF. Rather than functioning as a universal risk marker, galectin-3 emerges as a dynamic indicator of myocardial remodeling, particularly when assessed serially, capturing progressive fibrotic activity that is not fully reflected by natriuretic peptides or troponins. These findings refine the clinical positioning of galectin-3 from a static biomarker to a tool for longitudinal risk stratification and disease monitoring, while underscoring the need for phenotype-specific application. By integrating mechanistic plausibility with clinical evidence, this review advances the understanding of how galectin-3 can complement existing biomarkers and highlights its potential role in future precision-based approaches to HF management.

## References

[1] Nguyen AH, Hurwitz M, Abraham J (2023). Medical management and device-based therapies in chronic heart failure. J Soc Cardiovasc Angiogr Interv.

[2] Inam M, Sangrigoli RM, Ruppert L, Saiganesh P, Hamad EA (2025). Advancing heart failure care through disease management programs: a comprehensive framework to improve outcomes. J Cardiovasc Dev Dis.

[3] Cao Z, Jia Y, Zhu B (2019). BNP and NT-proBNP as diagnostic biomarkers for cardiac dysfunction in both clinical and forensic medicine. Int J Mol Sci.

[4] Tsutsui H, Albert NM, Coats AJ (2023). Natriuretic peptides: role in the diagnosis and management of heart failure: a scientific statement from the Heart Failure Association of the European Society of Cardiology, Heart Failure Society of America and Japanese Heart Failure Society. J Card Fail.

[5] Kanyal S, Das A, Bashir AM (2025). N-terminal pro-B-type natriuretic peptide (NT-proBNP) as a biomarker in heart failure with preserved ejection fraction (HFpEF) versus heart failure with reduced ejection fraction (HFrEF): the way forward in the age of proteomics. Cureus.

[6] Dong R, Zhang M, Hu Q, Zheng S, Soh A, Zheng Y, Yuan H (2018). Galectin-3 as a novel biomarker for disease diagnosis and a target for therapy (review). Int J Mol Med.

[7] Hara A, Niwa M, Kanayama T (2020). Galectin- 3: a potential prognostic and diagnostic marker for heart disease and detection of early stage pathology. Biomolecules.

[8] Zaborska B, Sikora-Frąc M, Smarż K, Pilichowska-Paszkiet E, Budaj A, Sitkiewicz D, Sygitowicz G (2023). The role of galectin-3 in heart failure: the diagnostic, prognostic and therapeutic potential: where do we stand?. Int J Mol Sci.

[9] Page MJ, McKenzie JE, Bossuyt PM (2021). The PRISMA 2020 statement: an updated guideline for reporting systematic reviews. BMJ.

[10] Brown D (2020). A review of the PubMed PICO tool: using evidence-based practice in health education. Health Promot Pract.

[11] Stang A (2010). Critical evaluation of the Newcastle-Ottawa scale for the assessment of the quality of nonrandomized studies in meta-analyses. Eur J Epidemiol.

[12] Nejadghaderi SA, Balibegloo M, Rezaei N (2024). The Cochrane risk of bias assessment tool 2 (RoB 2) versus the original RoB: a perspective on the pros and cons. Health Sci Rep.

[13] Ho JE, Liu C, Lyass A (2012). Galectin-3, a marker of cardiac fibrosis, predicts incident heart failure in the community. J Am Coll Cardiol.

[14] Edelmann F, Holzendorf V, Wachter R (2015). Galectin-3 in patients with heart failure with preserved ejection fraction: results from the Aldo-DHF trial. Eur J Heart Fail.

[15] French B, Wang L, Ky B (2016). Prognostic value of galectin-3 for adverse outcomes in chronic heart failure. J Card Fail.

[16] Felker GM, Fiuzat M, Shaw LK (2012). Galectin-3 in ambulatory patients with heart failure: results from the HF-ACTION study. Circ Heart Fail.

[17] Meijers WC, Januzzi JL, deFilippi C, Adourian AS, Shah SJ, van Veldhuisen DJ, de Boer RA (2014). Elevated plasma galectin-3 is associated with near-term rehospitalization in heart failure: a pooled analysis of 3 clinical trials. Am Heart J.

[18] van Vark LC, Lesman-Leegte I, Baart SJ (2017). Prognostic value of serial galectin-3 measurements in patients with acute heart failure. J Am Heart Assoc.

[19] Shah RV, Chen-Tournoux AA, Picard MH, van Kimmenade RR, Januzzi JL (2010). Galectin-3, cardiac structure and function, and long-term mortality in patients with acutely decompensated heart failure. Eur J Heart Fail.

[20] Min SH, Kim I, An JN, Lee HS, Kim SG, Kim JK (2025). Galectin-3 as a prognostic biomarker in haemodialysis patients with preserved or mildly reduced ejection fraction. Clin Kidney J.

[21] Liu T, Yang F (2025). Galectin-3 in acute myocardial infarction: from molecular mechanisms to clinical translation. Front Mol Biosci.

[22] Schupp T, Rusnak J, Forner J (2023). Cardiac troponin I but not N-terminal pro-B-type natriuretic peptide predicts outcomes in cardiogenic shock. J Pers Med.

[23] Coburn E, Frishman W (2014). Comprehensive review of the prognostic value of galectin-3 in heart failure. Cardiol Rev.

[24] Martuszewski A, Paluszkiewicz P, Poręba R, Gać P (2025). Galectin-3 in cardiovascular health: a narrative review based on Life's Essential 8 and Life's Simple 7 frameworks. Curr Issues Mol Biol.

[25] Mitić B, Jovanović A, Nikolić VN (2022). Trend of galectin-3 levels in patients with non-ST-elevation and ST-elevation myocardial infarction. Medicina (Kaunas).

[26] Keng BM, Gao F, Ewe SH (2019). Galectin-3 as a candidate upstream biomarker for quantifying risks of myocardial ageing. ESC Heart Fail.

[27] Bui TT, Dinh NH (2023). Galectin-3 changes from admission to discharge and its prognostic value for in-hospital mortality in heart failure: a prospective observational study. Medicine (Baltimore).

